# Highly Pathogenic Avian Influenza (H5N1) Clade 2.3.4.4b in Cattle: A Rising One Health Concern

**DOI:** 10.3390/ani15131963

**Published:** 2025-07-03

**Authors:** Ivan Camilo Sanchez-Rojas, D. Katterine Bonilla-Aldana, Catherin Lorena Solarte-Jimenez, Jorge Luis Bonilla-Aldana, Jaime David Acosta-España, Alfonso J. Rodriguez-Morales

**Affiliations:** 1 Grupo de Investigación en Recursos Naturales Amazónicos GRAM, Instituto Tecnológico del Putumayo, Mocoa 860001, Colombia; ivan.sanchez@itp.edu.co (I.C.S.-R.); catherin9420@gmail.com (C.L.S.-J.); 2 College of Medicine, Korea University, Seoul 02841, Republic of Korea; dbonilla@korea.ac.kr; 3 Grupo de Virologia, Universidad El Bosque, Bogotá 111321, Colombia; jorg.bonilla@udla.edu.co; 4 School of Medicine, Pontificia Universidad Católica del Ecuador, Quito 170525, Ecuador; 5 Health Sciences Faculty, Universidad Internacional SEK (UISEK), Quito 170120, Ecuador; 6 Institute of Microbiology, Friedrich Schiller University Jena, 07743 Jena, Germany; 7 Research Group of Emerging and Neglected Diseases, Ecoepidemiology and Biodiversity, Health Sciences Faculty, Universidad Internacional SEK (UISEK), Quito 170120, Ecuador; 8 Faculty of Health Sciences, Universidad Científica del Sur, Lima 15307, Peru; 9 Grupo de Investigación Biomedicina, Faculty of Medicine, Fundación Universitaria Autónoma de las Américas-Institución Universitaria Visión de las Américas, Pereira 660003, Colombia

**Keywords:** H5N1, cattle, highly pathogenic avian influenza, zoonosis, bovine, One Health

## Abstract

Highly pathogenic avian influenza (HPAI) H5N1, particularly clade 2.3.4.4b, has demonstrated an unprecedented capacity for cross-species transmission, with recent reports confirming its presence in dairy cattle in the United States of America (USA) in 2024. This unexpected spillover challenges traditional understanding of the virus’s host range and raises serious public health and veterinary concerns. Infected cattle presented with clinical signs such as decreased milk production, thickened or discolored milk, respiratory issues, and lethargy. Pathological findings revealed inflammation of the mammary glands and the detection of a virus in nasal secretions and raw milk, suggesting a potential for both intra- and interspecies transmission. While the current risk of human-to-human transmission remains low, the detection of H5N1 in a human exposed to infected cattle highlights the need for heightened surveillance and protective measures. Moreover, the presence of infectious viruses in the food chain, particularly in unpasteurized milk, introduces a new dimension of zoonotic risk. This review synthesizes emerging evidence on the epidemiology, pathology, diagnostic findings, and zoonotic implications of HPAI H5N1 infection in cattle. It also highlights the importance of genomic surveillance, intersectoral collaboration, and One Health approaches in managing this evolving threat. As the virus continues to circulate and adapt across diverse hosts, including wild birds, domestic poultry, and now mammals, the potential for reassortment and emergence of novel strains remains a significant concern. Immediate actions to strengthen biosecurity, monitor viral evolution, and protect both animal and human populations are critical to mitigate the global risk posed by this expanding panzootic.

## Simple Summary

Highly pathogenic avian influenza (H5N1), previously thought to affect birds primarily, has recently been detected in dairy cattle in the United States of America (USA). Infected cows exhibited signs such as reduced milk production, abnormal milk, and respiratory issues. The virus was also detected in raw milk, and a human case was linked to exposure to cattle. This unexpected spread raises new concerns for animal and human health. This review highlights what is currently known about the outbreak and emphasizes the urgent need for surveillance, biosecurity, and a One Health approach to prevent further spread of the disease.

## 1. Introduction

The influenza virus belongs to the family *Orthomyxoviridae* and is classified into four types: A, B, C, and D. In particular, the influenza A virus presents a wide variety of combinations between two surface proteins, hemagglutinin (H) and neuraminidase (N), with 18 known subtypes of H and 11 of N [1,2]. However, the five subtypes of most significant epidemiological relevance are H1N1, H2N2, H3N2, H5N1, and H7N9 [3,4,5,6].

Influenza A has been responsible for most of the pandemics documented in human history [7], including the Spanish flu (H1N1) in 1918, the Asian flu (H2N2) in 1957, the Hong Kong flu (H3N2) in 1968 [8], and swine flu (H1N1), the result of a mutation of the original virus, in 2009 [9]. The first outbreaks of avian influenza caused by the H5N1 subtype were recorded in poultry in Scotland, United Kingdom (UK), in 1959 [10] and geese in Guangdong Province, China, in 1996 [11,12].

It has been reported that the avian influenza virus is classified into two groups based on the intravenous pathogenicity index (IVPI), with highly pathogenic avian influenza (HPAI) and low pathogenic avian influenza (LPAI) [10,13,14]. In particular, the avian influenza virus subtype H5N1 is considered highly pathogenic (HPAI) [15], its main reservoir being the migratory birds of the order Anseriformes (e.g., geese, swans, ducks) and Chatradriformes (e.g., gulls, terns) [16]. In addition, H5N1 is characterized as having several clades, or genetic subdivisions; clade 2.3.4.4b is the one that has been recognized since 2016 [17].

**Figure 1:**
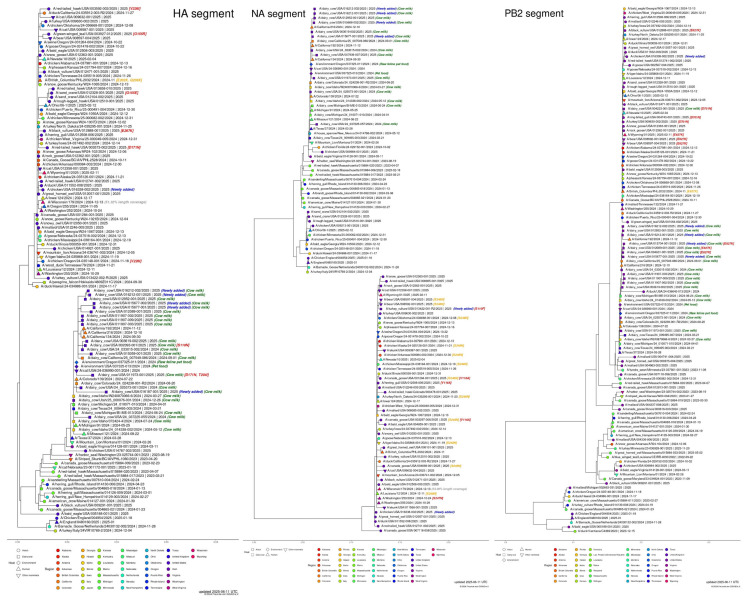
H5N1 bird flu in the USA, according to GISAID, 11 June 2025. (https://gisaid.org/resources/gisaid-in-the-news/highly-pathogenic-avian-influenza-outbreak-in-the-united-states/#c5130 (accessed 11 June 2025)). Subsampled phylogenetic trees with focus on recent USA H5N1 samples shown for HA, NA, and PB2 (as of 11 June 2025). Clade 2.3.4.4b of the highly pathogenic avian influenza (HPAI) virus, which has been responsible for outbreaks among wild and domestic birds globally, continues to spread among dairy cattle, poultry, and other animal species in the USA. Since April 2024, the USA CDC has confirmed 70 human infections based on genomic sequencing. In three of these cases, the NA-S247N amino acid substitution was detected, which may slightly reduce the virus’s sensitivity to the neuraminidase inhibitor oseltamivir in laboratory settings. Additionally, the CDC reported a separate mutation in the polymerase acidic (PA) protein in a virus isolated from a recently confirmed human H5N1 case in California.

Clade 2.3.4.4b spread globally through migratory birds; however, initial outbreaks and mortalities were mainly limited to avian species, including poultry [17]. HPAI H5N1 clade 2.3.4.4b is currently distributed in Europe, Africa, Asia, North America (including the United States of America [USA] and Canada), and South America (comprising Argentina, Bolivia, Brazil, Chile, Colombia, Ecuador, Peru, Uruguay, and Venezuela) (Figure 1) [18,19,20,21,22,23]. The mutation has allowed the virus to adapt to various conditions and spill over to multiple species, including humans [24], and it is responsible for the unusual outbreaks that have affected more than 43 species of mammals, such as minks, seals, rodents, opossums, raccoons, lions, cats, goats, and, of great concern, cattle [15,16,19,25,26,27,28,29,30]. Similarly, it has raised alarms due to its zoonotic potential after it was isolated as a causal agent of infection in humans [31,32].

**Figure 2:**
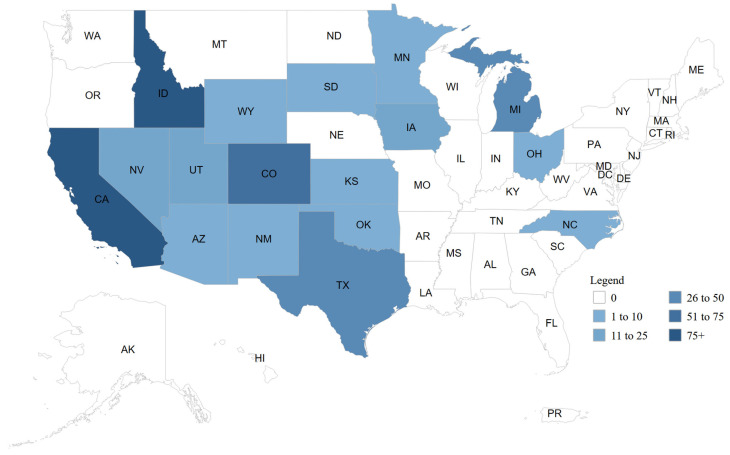
Number of confirmed cases of H5N1 in cattle by state, total outbreak, USA, March 2024–June 2025, according to the animal and plant health inspection service (APHIS) of the USA Department of Agriculture (USDA). Reproduced from https://www.aphis.usda.gov/livestock-poultry-disease/avian/avian-influenza/hpai-detections/hpai-confirmed-cases-livestock (accessed 11 June 2025).

In 2024, an outbreak of HPAI H5N1 clade 2.3.4.4b started in dairy cows in the USA (Figure 2) [33,34,35,36,37,38,39]. This event is considered both concerning and unusual, given that cows are routinely infected with influenza viruses. Still, when they do, the type involved is typically D. Therefore, this is the first time a highly pathogenic strain has been identified in this animal species [40]. Although this pattern initially suggested rapid spread, it is also possible that limited early surveillance delayed detection, and that infections may have occurred earlier, reflecting an underestimation of the actual timeline rather than accelerated transmission. However, although the first global report of an outbreak in cows occurred in 2024 in the USA, evidence of cattle susceptibility to HPAI H5N1 has been present since 2008, as shown in experimental research conducted by Kalthoff et al. [41]. The virus was inoculated in four calves, and although the animals remained healthy, seroconversion occurred in all of them, thus demonstrating that the virus had the potential to infect bovine calves.

Since the first notification of HPAI H5N1 infection in dairy cows in Texas, USA, in March 2024, rapid spread has occurred to more than 380 farms in 14 states [42]. It was determined that the infected animals cohabited or were close to poultry or wild bird populations [34]. It has also been suggested that cow-to-cow transmission could have occurred through respiratory fluids [39]. The clinical manifestations developed by infected cows were fever, lethargy, loss of appetite, runny nose, mastitis, decreased milk production, thickening of milk, reduced rumination, and changes in fecal consistency [28]. This outbreak has caused substantial economic losses for the livestock sector due to the decrease in the productive and reproductive parameters of the animals [19,35].

**Figure 3:**
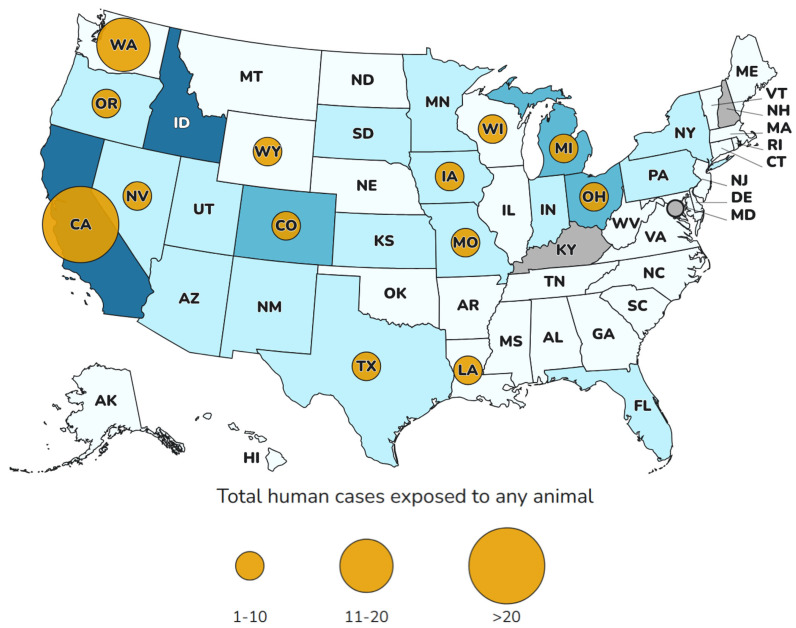
Influenza H5 virus infections in humans exposed to affected animals by state (1 January 2024–6 June 2025), according to the Centers for Disease Control and Prevention (CDC). Reproduced from https://www.cdc.gov/bird-flu/h5-monitoring/index.html (accessed 11 June 2025).

Cases of human infection with HPAI H5N1 in the USA have raised multiple questions due to the zoonotic characteristics of the virus (Figure 3) [43]. In August 2024, the country reported 14 cases of sick people, whose symptoms ranged from conjunctivitis to mild or moderate respiratory injuries, and they also reported having had previous exposure to cattle [44]. In December of that year, the figure rose to 59 people [45]. By January 2025, the Centers for Disease Control and Prevention (CDC) reported that a total of 67 confirmed cases of H5N1 avian influenza in humans were registered in the USA, with the news that in the state of Louisiana the death of a person previously hospitalized with a severe case of avian influenza (H5N1) had been reported, becoming the first fatal human case in the country (Figure 3) [46].

The current panzootic caused by the HPAI H5N1 virus that has infected cattle in the USA could become one of the most important due to its geographic range, number of infected animal species, zoonotic risk, and significant economic losses [47]. Although to date the transmission of the virus from person to person has not been proven, the characteristics of genetic mutation and ecological adaptation exhibited by the virus have made it an agent of great concern due to its pandemic potential [48].

The outbreak reported in cattle has generated multiple concerns about the imminent health risks that loom over the human population, as this virus has been detected in unpasteurized milk and wastewater, potentially threatening to enter the food chain [49,50]. Highly pathogenic avian influenza (HPAI) H5N1 underscores the need for One Health approaches, which necessitate multisectoral collaborations involving entities at the municipal, departmental, national, and international levels [51]. In addition to the above, cattle farming is one of the most critical livestock activities worldwide, which increases the risk of zoonoses to humans, either by direct exposure of workers on farms with infected animals or by the consumption of unpasteurized milk and derived dairy products [52]. In this article, based on the search of articles in the PubMed, Scopus, and Web of Science databases, mainly in the last four decades (1985–2025), we discuss the zoonotic, epidemiological, ecological, and clinical aspects, as well as the origin, distribution, and nature of the avian influenza (H5N1) virus in cattle.

## 2. Nature and Classification of Influenzavirus

The National Center for Biotechnology Information (NCBI) proposes that the taxonomy of influenza viruses is classified as Domain: Riboviria; Kingdom: Orthornavirae; Phylum: Negarnaviricota; Class: Insthoviricetes; Order: Articulavirales, and Family: Orthomyxoviridae (NCBI:txid11320) [53]. Regarding the genera, five were initially described within this family: *Influenzavirus A*, *Influenzavirus B*, *Influenzavirus C*, *Thogotovirus*, and *Isavirus* [54].

**Figure 4:**
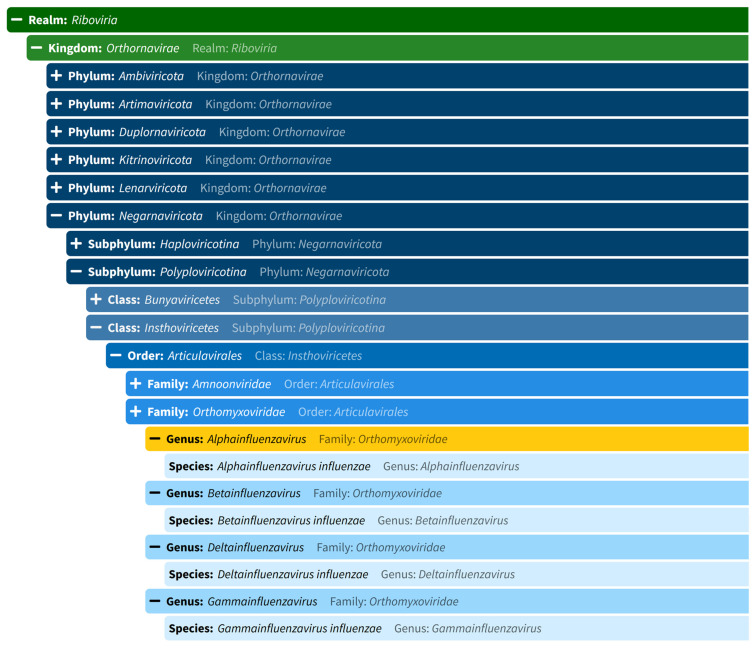
Taxonomic classification of the influenza viruses, according to the International Committee on Viral Taxonomy (ICTV), https://ictv.global/taxonomy (accessed 12 June 2025).

Today, the virus is recognized as *Alpha-influenzavirus* (influenza A virus), *Beta-influenza* (influenza B virus), *Gamma-influenza* (influenza C virus), and *Delta-influenza* (influenza D virus) (Figure 4) [1]. Influenza A and B viruses have eight segments of negative-sense RNA with two major surface glycoproteins, hemagglutinin (HA) and neuraminidase (NA), whereas influenza viruses C and D consist of seven segments of negative-sense RNA with only one major surface glycoprotein, hemagglutinin-esterase-fusion (HEF) [55].

The influenza D virus causes respiratory disease in pigs and ruminants; the influenza C virus colonizes the upper respiratory tract and induces influenza mainly in children; the influenza B virus produces respiratory symptoms having tropism through the upper and lower respiratory tract in adults; and the influenza A virus, whose symptoms are similar to B, is of global concern to health authorities since the antigenic drift of its main glycoprotein (hemagglutinin) allows it to mutate easily, adapting to various ecological and host contexts (humans and animals) [56].

**Figure 5:**
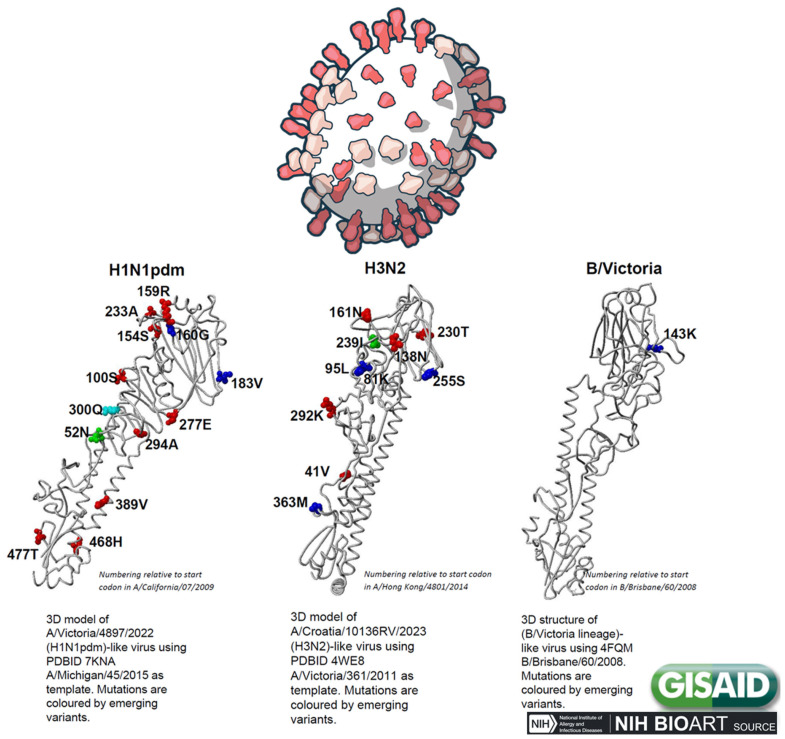
Changes in hemagglutinin for the top emerging variants based on sequences collected in the past 100 days. Influenza hemagglutinin mutation surveillance, EpiFlu update, January 2025 (www.gisaid.org (accessed 11 June 2025)).

Influenza A viruses encode at least ten major proteins: hemagglutinin (HA) (Figure 5), basic polymerase 1 and 2 (PB1-PB2), acid polymerase (PA), nucleoprotein (NP), neuraminidase (NA), matrix 1 and 2 (M1-M2), nonstructural protein 1 and 2 (NS1-NS2), nuclear export protein (NEP), and others with specific or as yet unknown functions such as PB1-F2, PB1-N40, PA-X, and PA-N155 and PA-N182 [57].

The replication cycle of this virus is a complex process [58,59]. It begins when the virus attaches to the host cell via hemagglutinin (HA), which interacts with sialic acid receptors on the cell surface [60]. After the virus enters through endocytosis, the low pH in the endosome facilitates the fusion of the viral envelope with the endosome membrane, releasing the viral genome into the cytoplasm [61]. The genetic material is transported to the nucleus where the viral RNA polymerase initiates the transcription and replication of the viral RNA; transcription generates positive-sense mRNA for viral protein synthesis in the cytoplasm and complementary RNA (cRNA) that will serve as a template for new copies of viral genomics RNA (vRNA) [62]. The viral proteins hemagglutinin (HA), neuraminidase (NA), and Matrix 2 (M2) are synthesized in the endoplasmic reticulum and then transported to the Golgi apparatus, where they are directed to the plasma membrane [1].

The assembly of the virion occurs near the plasma membrane, where the viral ribonucleoprotein complex (vRNP) and structural proteins bind to the cell’s plasma membrane to use it as an envelope, while neuraminidase facilitates the release of viral particles by cutting the sialic acid bonds between the virus and the host cell [63]. As a result, new viral particles are released by budding and can infect other cells, starting a new cycle of infection [64].

The key factor in virus entry into an organism is the hemagglutinin surface glycoprotein HA (strongly expressed in influenza A virus), which contains the host receptor binding site to allow the virus particle to attach to specific host cells, the fusion peptide that is inserted into the target cell membrane during membrane fusion, and other structural elements that can retract during the membrane fusion process [65]. Since HA is a surface glycoprotein of the virus particle, it is easily recognized by antibodies; however, the virus can evade the host’s immune system through unique mechanisms. During its replication, viral RNA polymerase makes frequent mistakes, allowing the creation of HA variants that will allow the virus to become resistant to existing antibodies, facilitating immune system evasion and virus replication in the infected host [65,66].

Undoubtedly, influenza A viruses, which can cause severe pneumonia and ultimately lead to death, pose a significant threat to human health, as such viruses can recombine segments, resulting in the emergence of pandemic strains for which there is little pre-existing immunity in the human population [55,67,68]. This condition of mutation has allowed influenza A virus to be the virus with the highest number of pandemics in the history of humanity, as well as being responsible for significant human mortality figures in recent times [8], as in the case of the Spanish flu of 1918, which left around 21 million people dead [69], surpassing even COVID-19, which as of February 2025 has registered more than 7 million deaths [70].

Within the set of influenza A viruses, the H5, H6, H7, H9, and H10 subtypes are associated with avian influenza, which is distinguished from seasonal/pandemic influenza or influenza (H1N1, H3N2) because the former may have highly pathogenic strains that are capable of inducing greater damage to the organs and systems of the body, and is therefore a more serious disease in animals and humans [10,15]. The H5N1 strain of highly pathogenic avian influenza (HPAI) shows a basic reproductive index (*R0*) which varies between 0.7–2.68 [71,72], as well as a prevalence of 58–84% in the human population exposed to poultry. Additionally, HPAI H5N1 and HPAI H7N9 show higher mortality rates than other subtypes such as H1N1, H2N2, and H3N2 [3]. In the same way, the evidence of HPAI H5N1’s ability to adapt and mutate is striking and indicates that this virus has evolved in recent years: in 2008 clade 2.3.4.4 was registered, and since 2016 another genetic subdivision has been isolated, clade 2.3.4.4b [17]. Also, it is known that before 2020, the neuraminidase (NA) protein of subtype N1 of clade 2.3.4.4b had a truncated peduncular domain that mediated virulence factors in poultry; recently, with new infections, it has been possible to determine that the vast majority of highly pathogenic H5N1 viruses of clade 2.3.4.4b currently circulating worldwide have the extended peduncular version of neuraminidase, which may increase the risk of these viruses being transmitted between humans [73].

## 3. Worldwide Distribution of H5N1

Historically, since its first appearances in Scotland (UK) and China in 1959 and 1996, respectively, H5N1 HPAI had been limited to poultry; however, mutations have allowed the virus to adapt and infect wild birds [10,11,49]. This situation has led to an increasing number of infections in waterfowl, raptors, and peridomestic species in recent years [16]. The rapid intercontinental spread of the virus, facilitated by the migration of infected birds, the genomic rearrangements of the virus, and the unusual spread in terrestrial and marine mammals, has triggered a panzootic of HPAI H5N1 and raised significant pandemic risk concerns [7]. More than two decades after HPAI H5N1 became established in poultry in Southeast Asia, the virus has spread to various countries in Africa, South America, North America, and Europe, and to Antarctica [74,75,76,77,78,79,80,81,82,83,84,85,86,87,88,89,90,91,92].

As previously reported, the HPAI H5N1 virus is distributed globally and has infected multiple animal species [93]. In Chile, important implications in wild birds were reported with the death of Peruvian pelicans (*Pelecanus thagus*), Franklin’s gulls (*Larus pipixcan*), grey gulls (*Leucophaeus modestus*), elegant terns (*Thalasseus elegans*), and black graters (*Rynchops niger*) [94,95]. In Peru, there have been mass deaths of common dolphins (*Delphinus delphis*) and South American sea lions (*Otaria flavescens*) [81]. Also in Peru, a case was documented in neotropical cormorants (*Nannopterum brasilianum*) and a lion (*Panthera leo*) [15]. In Punta Bermeja, the largest colony of South American sea lions in Argentina, more than 800 animals of this species died [21]. Also, rodents *Rattus norvegicus* and *Rattus rattus* were found to be HIV-positive in the Giza region, Egypt [96].

**Figure 6:**
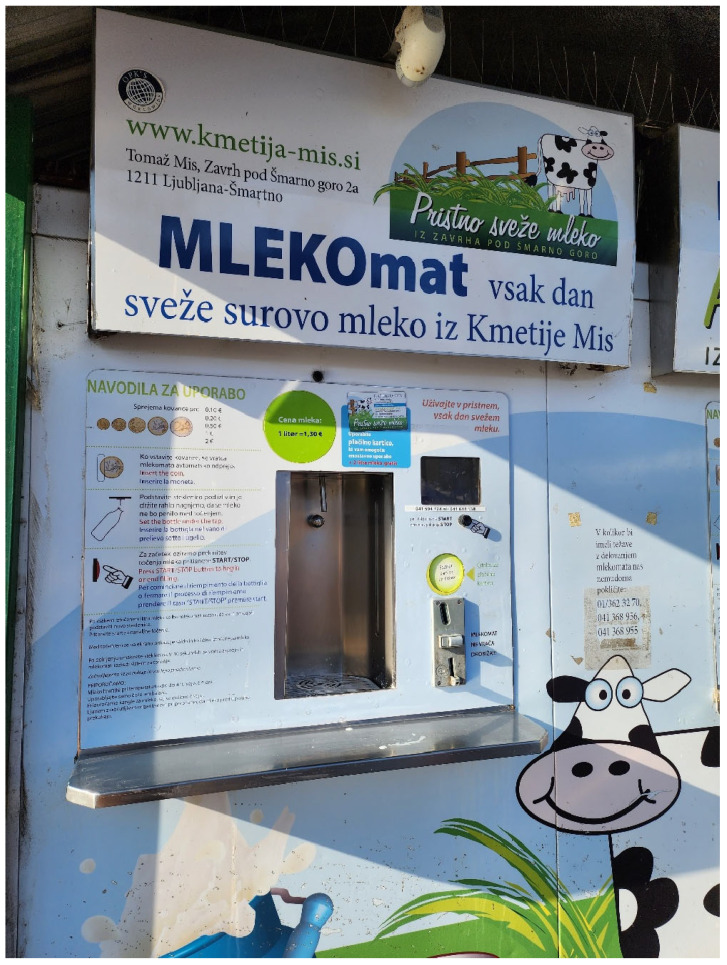
Fresh raw milk vending machine in Ljubljana, Slovenia. Photo taken by AJRM on 28 January 2024. The text says: MLEKOmat—every day fresh raw milk (stored at 4 °C up to three days) from the Miš farm. Logo: “Authentic fresh milk from Zavrh under Šmarna gora”. The price of a liter is 1.30 euros. A note at the bottom left says: “Note: Use clean bottles only! Milk must be boiled before use!” (https://www.mleko-mat.si/mlekomat/ (accessed 11 June 2025)).

Regarding domestic mammals, there is a history of HPAI H5N1 seropositivity in cats, dogs, and, more recently, cattle [28,97]. In Poland, a case was registered of a male cat, neutered, approximately 6 years old, with a history of feeding on raw chicken meat, who was taken to a clinic for signs of apathy and anorexia that, after three days, evolved into respiratory distress and neurological signs that led to the death of the animal [98]. In France, the virus was detected in a domestic cat that lived near a duck farm [99]. In South Dakota, USA, 10 cats died, whose postmortem studies reflected lesions and viral antigens in different organs, mainly in the brain [100]. Similarly, on a dairy farm in the USA, cats of different ages died after being fed raw milk from cows [17]. Curiously, some countries (e.g., Slovenia) still sell fresh raw milk in public street vending machines (Figure 6).

In addition to the limited clinical signs reported in infected dairy cattle—such as reduced milk production and transient fever—recent studies have begun to characterize the pathological changes associated with HPAI H5N1 clade 2.3.4.4b infection in this novel host [42]. Gross lesions in affected cattle have included pulmonary consolidation, interstitial pneumonia, and evidence of encephalitis on necropsy. Histopathological evaluations have revealed interstitial pneumonia with lymphohistiocytic infiltration, multifocal hepatic necrosis, and nonsuppurative encephalitis [101,102]. These findings suggest a broader systemic involvement of the virus in bovines than initially anticipated. Notably, viral RNA and antigens have been detected in multiple organs, including the brain, lungs, and mammary tissue, supporting the neurotropic and pneumotropic properties of the virus. This pathological profile complements the emerging understanding of the multisystemic nature of H5N1 clade 2.3.4.4b infection across mammalian hosts, including domestic cats [26,100].

Concerns about H5N1 transmission in cats focus on the consumption of infected birds and contaminated milk; however, the anthropozoonotic risk is often overlooked. In Michigan, USA, a cat that did not have access to the outside world tested positive for the virus and developed clinical manifestations with neurological deterioration. The owner reported that he worked on H5N1-positive dairy farms and transported unpasteurized milk, did not wear protective equipment, and that, when he arrived at the house, the cat rolled around in his clothes [103].

In Washington, USA, antibodies to HPAI H5N1 virus were detected in 4/194 (2%) dogs that hunted or participated in hunting and training trials with wild birds [104]. It was also shown that inoculation of the virus in beagle dogs resulted in excretion of the virus and rapid seroconversion without disease [105]. In an investigation carried out in China, it was found that after dogs were infected with the virus intranasally and intratracheally, they developed disease, including anorexia, fever, conjunctivitis, respiratory distress, and cough, and in some cases died [106].

An important and unusual aspect of HPAI H5N1 infection in pigs is that, although experimental infections have demonstrated interstitial pneumonia with necrotizing bronchiolitis, high viral titers in the lower respiratory tract, and 100% seroconversion, the infected animals shed only limited amounts of virus and do not transmit it through direct contact [107]. Due to these characteristics, pigs are considered potential intermediate hosts that can facilitate genetic reassortment of the virus, enabling interspecies transmission events that may contribute to the emergence of pandemic strains [108].

## 4. History of Influenza Virus Infection in Livestock

It should be noted that, although the first natural infection with influenza A virus in cattle was recorded during the 2024 outbreak, cases of natural infection with influenza D (IDV) had already been documented in these animals [109]. This virus was initially isolated in Oklahoma, USA, from a 15-week-old pig that showed influenza-like signs [110]. Later evidence showed that the IDV is more common in cattle than in pigs, which is why it is believed that cattle act as the main reservoir of the virus [55]. Although the initial detection occurred in pigs in 2011, serological surveillance suggests that IDV has been present in cattle since at least 2004 [111].

Calves and fattening cattle are often the most susceptible to IDV infection [111,112]. In recent years there has been increasing evidence supporting the theory that, along with the bovine viral diarrhea (BVDV), bovine herpesvirus 1 (BHV-1), the bovine respiratory syncytial virus (BRSV), and parainfluenza virus type 3 (PI3), the influenza D virus (IDV) is linked to the bovine respiratory disease complex [113]. However, findings of positive samples for IDV, not only from cattle showing clinical signs associated with bovine respiratory disease, but also from healthy cattle, would demonstrate that the virus can cause infection without clinical signs [114].

IDV has been distributed around the world and has been associated with livestock infections in several countries such as Brazil, China, France, Ireland, Italy, Japan, Luxembourg, and the USA [112,115,116,117,118]. In turn, in addition to cattle and pigs, IDV antibodies have been detected in goats, sheep, horses, camels, and buffalo [56,114]. Similarly, there is evidence of infection in exposed and non-exposed persons to livestock, indicating that influenza virus type D, like type A, has zoonotic potential [55].

## 5. H5N1 in Cattle

The outbreak of HPAI H5N1 in cattle in the USA in 2024 raised significant concerns regarding public health and food safety [50]. However, it is essential to note that the virus had already been detected in over 200 mammalian cases in the country since 2022, suggesting prior cross-species transmission events before its emergence in cattle [119]. These facts support the evolutionary line of HPAI H5N1 in the USA, as the virus has been detected in poultry and wild birds since its entry through the Canadian border in 2021, and subsequently in cats, goats, and cattle [19]. On 20 March 2024, the Minnesota Board of Animal Health reported that a kid raised on a farm in Stevens County, west-central Minnesota, tested positive for highly pathogenic avian influenza (HPAI); an outbreak of HPAI H5N1 in poultry had recently been detected on this same farm [120]. In turn, on 25 March 2024, the first outbreak in the world of HPAI H5N1 clade 2.3.4.4b virus was reported in dairy cows in three USA states (Texas, Kansas, and New Mexico). Subsequently, on 29 March 2024, the virus was detected in cattle from a farm in Michigan that had recently imported animals from Texas, and from this cases were reported in different states of the country [119,120,121,122,123,124]. Phylogenetic analysis of HPAI H5N1-positive samples, clade 2.3.4.4b, in dairy cattle indicated that the reassociation of the virus likely occurred in wild birds, followed by transmission to dairy cattle at the end of 2023 [125]. Following the introduction of the virus in cattle, its spread in the USA was facilitated by the movement of dairy cattle that showed no clinical signs but infected other animal species and humans [19].

In dairy cattle, HPAI H5N1 clade 2.3.4.4b infection presents with fever, lethargy, dehydration, loss of appetite, clear nasal discharge, increased breathing rate, difficulty breathing, reduced rumination, changes in stool consistency, such as diarrhea or dry stools, and involution of the mammary gland in several of the affected cows, in addition to a notable drop in milk production that can take on an abnormal yellowish color similar to colostrum, with a thick and sometimes curdled consistency [19,28,31,35,39]. In a study carried out by Caserta et al. [27], the virus was reported to have caused mortality in some infected cows. Meanwhile, cattle that managed to recover experienced a clinical illness that lasted between 5 and 14 days, but they continued to exhibit decreased milk production for at least 4 weeks.

One of the main determining factors in the virus–host relationship is the availability of receptors that allow viral development: in particular, influenza A viruses use sialic acids from the host as their receptors for the initial binding of the hemagglutinin protein (HA) and entry into cells. It has been reported that the respiratory and mammary glands of dairy cattle naturally infected in the USA with HPAI H5N1 are rich in avian influenza virus-specific sialic acid, suggesting virus epitheliotropism in the mammary glands of cattle [29,126]. It has also been observed that the bovine H5N1 virus replicates efficiently in the epithelium of the glandular cistern and teats of dairy cows, demonstrating that these viruses can invade the mammary gland through the teat canal [127].

Raw milk from infected animals has been reported to contain a high viral load, representing a potential source of dissemination and spread of the virus [128]. Although, in principle, milk from visibly sick cows is withheld from commercial distribution [40], recent reports revealed that milk sold in 10 U.S. states contained H5N1 viral fragments [35]. In addition, it has been found that the virus can remain infectious on fomites and milking equipment materials for several hours [129,130]. In addition, research has concluded that pasteurization is the only effective method to achieve viral inactivation of HPAI H5N1 in cattle milk [128,131]. However, it cannot be ignored that cattle infected during the outbreak in the USA also presented high viral titers in nasal swab samples; therefore, while it is true that milk and fomites are essential sources of infection, respiratory fluids also generate concern [39].

Something very striking is that two years before the detection of HPAI H5N1 in dairy cattle, the virus was already detected in wastewater in the USA, with no relation or presence of dairy processing facilities or dairy farms within the sewer basin [132]. Subsequently, in investigations carried out during the 2024 outbreak, the virus was again detected in wastewater, and this time the basin included milk processing sources [50].

Although the mechanism by which cattle were infected in the USA is unclear, official reports indicate that the farms where the outbreaks occurred had reported simultaneous mortality events in poultry and wild birds [26]. So far, the most reasonable explanation for HPAI H5N1 infection in cattle is related to contact with fluids or tissues from infected birds, as different studies have shown that the excretion of the virus in birds occurs through the oropharyngeal and cloacal pathways, with maximum expression in the oropharyngeal pathway [133,134,135]. On the other hand, the entry of the virus into mammals can occur nasally or oropharyngeally [41,136].

Recent studies have also identified farmed pigs as susceptible to infection with HPAI H5N1 clade 2.3.4.4b, raising concerns about their role as potential intermediate hosts or reservoirs [137,138]. Experimental and field investigations have reported the presence of viral RNA, seroconversion, and, in some cases, mild respiratory signs in pigs exposed to infected poultry or contaminated environments. Although clinical disease in swine has generally been subclinical or mild, the detection of H5N1 genetic material in porcine tissues and secretions underlines their possible role in sustaining or amplifying viral transmission. Given pigs’ capacity to support both avian and human influenza viruses, they represent a key node in interspecies transmission dynamics, particularly in mixed farming systems. These findings underscore the need to include pigs in integrated surveillance frameworks to better characterize the ecology of H5N1 across domestic animal populations and evaluate their role in potential zoonotic spillover events [139,140,141].

## 6. H5N1 in Humans

The first case of human HPAI H5N1 infection in the world was reported in the Hong Kong Special Administrative Region (China) in 1997. Since then, the virus reappeared in 2003 and continues to infect people around the world, posing a potential pandemic threat due to its continued global spread and evolution [142]. Although H5N1 has not adapted to human-to-human transmission in nearly thirty years, currently the abundant circulation in various animal species, including mammals, increases the possibility of recombination of new pandemic strains [143].

The World Health Organization (WHO) has reported a total of 954 cases of H5N1 infection in humans with 464 deaths, from January 2003 to December 2024 worldwide (Table 1). Cases have been reported in 24 countries, with Egypt (n = 359), Indonesia (n = 200), Vietnam (n = 129), Cambodia (n = 72), and in the USA (n = 59) being the countries with the highest number of cases, accounting for 85% of the total cases reported worldwide. Indonesia is the country with the highest mortality rate compared to its reported cases [45].

According to CDC figures, by 6 January 2025, the cumulative number of human HPAI H5N1 infections in the USA had risen from 59 to 67 cases, and the first death was reported [46]. It is estimated that most documented human infections in the USA resulted from dairy farm workers’ exposure to unpasteurized milk during milking [34,130,144,145]. People infected with HPAI H5N1 developed mild respiratory symptoms and conjunctivitis [44], or were asymptomatic, as in the case of three veterinarians with positive serological results for the highly pathogenic avian influenza A(H5) virus [146]. On the other hand, a case was reported of a 65-year-old deceased person with underlying medical conditions who, after being exposed to a flock of non-commercial backyard birds and wild birds, developed severe respiratory complications [147].

On 1 April 2024, the first case of suspected transmission of HPAI H5N1 virus from cow to human was reported in Texas, USA, after the CDC confirmed that a worker on a commercial dairy farm tested positive by real-time reverse transcriptase polymerase chain reaction (RT-qPCR) for HPAI H5N1 clade 2.3.4.4b infection. The patient only experienced conjunctivitis with no other signs or symptoms, was treated with oseltamivir, and recovered. No disease was identified among the patient’s household members, all of whom received post-exposure prophylaxis with the same antiviral [34].

Subsequently, in May 2024, two adult dairy farm workers in Michigan, USA, were identified as infected with the virus; one of these presented discomfort in his right eye a day after milk splashed in his eye while milking a cow, while the other worker, from another farm, presented cough, difficulty breathing, headache, sore throat, fatigue, nasal congestion, and rhinitis. The tasks of this second worker mainly included the care of sick cows, including administration of drugs orally; it should be mentioned that the worker used eye protection and gloves, but did not use a respirator or mask [148].

**Figure 7:**
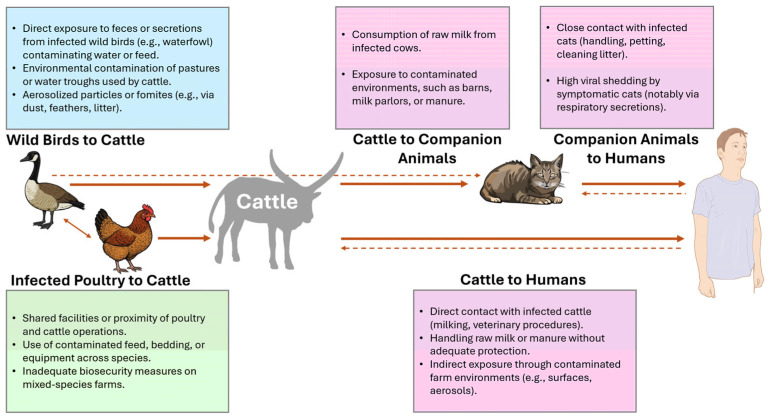
Proposed transmission routes of H5N1 to cattle and the possible spillover to humans. Developed using NIH BioArt (https://bioart.niaid.nih.gov/) and Biorender (https://app.biorender.com/) tools (accessed 11 June 2025). Arrows indicate the main route of transmission, and dashed arrows indicate potential theorical transmission.

Humans may contract H5N1 directly from cattle or via companion animals, especially cats that may have been infected by cattle (Figure 7).

Concerning the human immune response against HPAI H5N1, studies are limited; however, research has been carried out in animals, such as a study in ferrets, in which it was found that animals that were infected with H5N1 and had previously been exposed to the H1N1 virus had a cross-reaction with the neuraminidase (NA) protein of H5N1. This was evidenced by the low viral load present in nasal secretions and organs external to the respiratory tract, thus suggesting that humans with immunity to the H1N1 virus may experience milder illness due to the influenza A H5N1 virus strain [145]. Likewise, it has been reported that the H5N1 vaccines available in the USA, which are of a different strain and clade than the one presented in the 2024 outbreak, generate neutralizing crossover antibodies against H5N1 of the clade 2.3.4.4b circulating in humans and may be helpful as temporary alternative vaccines while a specific one for the emerging clade 2.3.4.4b is developed [33].

In this sense, the unprecedented intercontinental spread that since 2020 has been occurring with the highly pathogenic avian influenza (H5N1) virus to multiple species of animals including birds, mammals, and humans has generated alarm and concerns about public health due to its zoonotic and pandemic potential. Therefore, it is essential to continue implementing and strengthening research, international cooperation, and active surveillance of the virus in different hosts to establish prevention and control strategies to safeguard the world population. This should include H5N1 in cattle (Table 2) [149].

A comprehensive and multidisciplinary diagnostic framework is essential for a complete understanding of the complex ecology of HPAI H5N1 clade 2.3.4.4b infections, especially as they now span multiple species, including birds, domestic animals, wildlife, and humans. Investigating cases in isolation—by species or sector—risks overlooking critical epidemiological linkages and shared transmission pathways [51,150]. Therefore, an integrated One Health approach should be prioritized, incorporating simultaneous sampling and testing of animals (e.g., poultry, cattle, cats), human contacts, and environmental reservoirs. This includes harmonized surveillance protocols, shared genomic sequencing databases, and joint outbreak investigations. Only through such cross-sectoral coordination can effective and timely measures be implemented to characterize, prevent, and control this expanding threat, while mitigating its potentially devastating impact on both human and animal health [51,151,152].

## 7. Limitations

This review is limited by the rapidly evolving nature of the HPAI H5N1 outbreak in cattle, with much of the available data being preliminary and subject to revision. Many findings are based on case reports, early surveillance data, and experimental studies, which may not capture the full scope of viral transmission dynamics or clinical outcomes. Additionally, there is a limited amount of peer-reviewed literature specifically on bovine H5N1 infections, which constrains comprehensive analysis. Future studies with broader epidemiological and molecular data will be essential to validate and expand upon current observations.

## 8. Conclusions

The emergence of highly pathogenic avian influenza (HPAI) H5N1 in cattle, particularly the clade 2.3.4.4b strain identified in the USA in 2024, marks a concerning development in the epidemiology of zoonotic diseases. Historically restricted to avian species and certain mammals, H5N1’s confirmed ability to infect bovines underscores the virus’s expanding host range and adaptive capacity. Clinical evidence, including significant declines in milk production, mammary gland pathology, and respiratory distress, highlights the impact on livestock health and productivity. Moreover, the detection of infectious viral particles in raw milk and nasal secretions introduces potential risks to the food chain and public health.

Although human-to-human transmission remains unproven, confirmed human infections linked to exposure to infected cattle and unpasteurized milk demand heightened vigilance. The possibility of further interspecies transmission and viral reassortment elevates the risk of a future pandemic scenario. Given the significant economic losses and public health implications, coordinated surveillance, rapid diagnostics, and stringent biosecurity measures are imperative.

While the transmission of HPAI H5N1 clade 2.3.4.4b has predominantly been reported from birds to mammals, including cattle and humans, the virus’s growing host range and genetic adaptability raise concerns about potential bidirectional transmission pathways. Documented cases in domestic animals with no direct avian exposure—such as indoor cats exposed to contaminated human clothing—suggest that reverse zoonosis, although currently unconfirmed, is a plausible possibility. These observations underscore the need to consider anthropozoonotic risk as part of a comprehensive One Health approach. Enhanced genomic surveillance and host susceptibility studies across species are crucial for better understanding and anticipating the virus’s adaptive potential in diverse ecological contexts.

This situation calls for a robust One Health approach—integrating human, animal, and environmental health disciplines—to monitor and mitigate the ongoing panzootic threat. Continued genomic surveillance and international collaboration will be crucial for tracking viral evolution and adapting preventive strategies. In conclusion, the unprecedented spillover of HPAI H5N1 into cattle not only challenges current paradigms of influenza virus ecology but also highlights urgent gaps in preparedness and response capacities that must be addressed globally.

## Figures and Tables

**Table 1:** Number of confirmed human H5N1 cases and deaths worldwide during 2003–2024.

Country	2003–2009		2010–2014		2015–2019		2020		2021		2022		2023		2024		Total	
	Cases	Deaths	Cases	Deaths	Cases	Deaths	Cases	Deaths	Cases	Deaths	Cases	Deaths	Cases	Deaths	Cases	Deaths	Cases	Deaths
Australia	0	0	0	0	0	0	0	0	0	0	0	0	0	0	1	0	1	0
Azerbaijan	8	5	0	0	0	0	0	0	0	0	0	0	0	0	0	0	8	5
Bangladesh	1	0	6	1	1	0	0	0	0	0	0	0	0	0	0	0	8	1
Cambodia	9	7	47	30	0	0	0	0	0	0	0	0	6	4	10	2	72	43
Canada	0	0	1	1	0	0	0	0	0	0	0	0	0	0	1	0	2	1
Chile	0	0	0	0	0	0	0	0	0	0	0	0	1	0	0	0	1	0
China	38	25	9	5	6	1	0	0	0	0	1	1	1	0	1	0	56	32
Djibouti	1	0	0	0	0	0	0	0	0	0	0	0	0	0	0	0	1	0
Ecuador	0	0	0	0	0	0	0	0	0	0	1	0	0	0	0	0	1	0
Egypt	90	27	120	50	149	43	0	0	0	0	0	0	0	0	0	0	359	120
India	0	0	0	0	0	0	0	0	1	1	0	0	0	0	0	0	1	1
Indonesia	162	134	35	31	3	3	0	0	0	0	0	0	0	0	0	0	200	168
Iraq	3	2	0	0	0	0	0	0	0	0	0	0	0	0	0	0	3	2
Lao	2	2	0	0	0	0	1	0	0	0	0	0	0	0	0	0	3	2
Myanmar	1	0	0	0	0	0	0	0	0	0	0	0	0	0	0	0	1	0
Nepal	0	0	0	0	1	1	0	0	0	0	0	0	0	0	0	0	1	1
Nigeria	1	1	0	0	0	0	0	0	0	0	0	0	0	0	0	0	1	1
Pakistan	3	1	0	0	0	0	0	0	0	0	0	0	0	0	0	0	3	1
Spain	0	0	0	0	0	0	0	0	0	0	2	0	0	0	0	0	2	0
Thailand	25	17	0	0	0	0	0	0	0	0	0	0	0	0	0	0	25	17
Turkey	12	4	0	0	0	0	0	0	0	0	0	0	0	0	0	0	12	4
UK	0	0	0	0	0	0	0	0	1	0	0	0	4	0	0	0	5	0
USA	0	0	0	0	0	0	0	0	0	0	1	0	0	0	58	0	59	0
Vietnam	112	57	15	7	0	0	0	0	0	0	1	0	0	0	1	1	129	65
Total	468	282	233	125	160	48	1	0	2	1	6	1	12	4	72	3	954	464

Source: World Health Organization (https://www.who.int/publications/m/item/cumulative-number-of-confirmed-human-cases-for-avian-influenza-a(H5N1)-reported-to-who--2003-2024--20-december-2024 (accessed 11 June 2025)).

**Table 2:** Challenges and strategies in surveillance, diagnostics, control, and prevention of H5N1 in cattle.

Domain	Challenges	Strategies
Surveillance	Lack of routine influenza monitoring in cattle Subclinical or mild cases may go unnoticed Weak integration across human-animal-environment sectors	Implement active surveillance in dairy farms, especially in high-risk regions Promote One Health data-sharing platforms Apply syndromic surveillance (e.g., milk production drop, respiratory signs)
Diagnostics	Few validated diagnostic tools for H5N1 in bovines Interpretation difficulties in infections without clinical signs Delays in sample collection, transport, and testing	Develop and validate specific assays (e.g., RT-PCR, ELISA) for cattle Train veterinarians and technicians on H5N1 testing protocols Expand access to regional diagnostic labs
Control	Difficulty isolating infected animals in large herds Gaps in farm-level biosecurity Possible environmental or wildlife reservoirs	Strengthen on-farm biosecurity and hygiene protocols Enforce targeted animal movement restrictions Minimize contact between cattle and wild birds or mammals
Prevention	No licensed vaccines for H5N1 in cattle Low awareness of zoonotic risks among farmworkers Contamination risk via raw milk	Support vaccine development and regulatory evaluation Educate workers on PPE use and hygiene Promote pasteurization and restrict raw milk distribution in affected areas

## Data Availability

Available upon reasonable request.
